# Partitioning of Fungal Endophyte Assemblages in Root-Parasitic Plant *Cynomorium songaricum* and Its Host *Nitraria tangutorum*

**DOI:** 10.3389/fmicb.2018.00666

**Published:** 2018-04-05

**Authors:** Jin-Long Cui, Vinod Vijayakumar, Gang Zhang

**Affiliations:** ^1^Institute of Applied Chemistry, Shanxi University, Taiyuan, China; ^2^Department of Food Science and Technology, College of Food, Agricultural, and Environmental Sciences, The Ohio State University, Columbus, OH, United States; ^3^College of Pharmacy, Shaanxi University of Chinese Medicine, Xianyang, China

**Keywords:** parasitic plant, plant-fungus interaction, *C. songaricum*, N. tangutorum, endophytic fungi, tripartite symbiosis

## Abstract

Endophytic fungi are an integral part and even seen as host organs of plant, influencing physiology, ecology, and development of host plants. However, little is known about micro-ecosystems and functional interactions of endophytic fungi in root-parasitic interactions of *Cynomorium songaricum* and its host *Nitraria tangutorum*. Here, distribution and dynamics of endophytic fungi were objectively investigated in their associations with *C. songaricum* and *N. tangutorum* based on mycobiome studies using high-throughput sequencing. Results suggest that endophytic fungi may be exchanged between *C. songaricum* and its host *N. tangutorum* probably through haustorium, connection of xylem and phloem in the vascular system. The similarity of endophytic fungal composition between *C. songaricum* and parasitized *N. tangutorum* was 3.88% which was significantly higher than the fungal similarity of 0.10% observed between *C. songaricum* and non-parasitized *N. tangutorum*. The similarities of fungal community in parasitized *N. tangutorum* were much closer to *C. songaricum* than to the non-parasitized *N. tangutorum*. The composition of endophytic fungi in these associations increased in progressive developmental stages of *C. songaricum* from sprouting to above ground emergence, and decreased subsequently probably due to host recognition and response by fungi. However, the shared fungal operational taxonomic units (OTUs) increased among interactions of *C. songaricum* with parasitized and non-parasitized *N. tangutorum*. Studies of bioactivity on culturable endophytic fungi showed that isolates such as *Fusarium* spp. possess the ability to promote seed germination of *C. songaricum.* Our study reports for the first time the special ecological system of endophytic fungi in *C. songaricum* and its host *N. tangutorum*. Overall, we hypothesize that a deeper understanding of the sharing, movement, and role of endophytic fungi between root-parasitic plant and its host may lead to finding alternative approaches to help increase the output of ethno-pharmacologically important medicinal plants.

## Introduction

*Cynomorium songaricum* Rupr., is a rare medicinal herb plant with pharmacological activities of treating various sexual conditions such as premature ejaculation, low sexual function, impotence, and spermatorrhea colic (Cui et al., 2013; Lee et al., 2013). It is often found parasitizing the roots of several plant families such as Nitrariaceae, Tamaricaceae, and Chenopodiaceous shrubs located in dry, rocky, or sandy soils of extreme desert environments (Cui et al., 2013). In China, *Nitraria tangutorum* and *Nitraria sibirica* of the Nitrariaceae are the preferred hosts of *C. songaricum*, and the flowering-fruiting season for *C. songaricum* has been described to be from May to August (Wan and Chen, 2000). *C. songaricum* parasitizing *N. tangutorum* forms a successfully evolving sustainable ecosystem in which mature seeds are dispersed with sand under natural desert conditions. Occasionally, a few seeds encounter the roots of *N. tangutorum*, invading plants using host root-secreted hormone, strigolactones (Kruh et al., 2017). The seed then begins to develop special intrusive organs such as haustorium which directly connects to the vascular system of host under suitable spatial and temporal conditions. Because *C. songaricum* plant has no chlorophyll and is unable to photosynthesize, it lives as a holoparasite, i.e., totally dependent on its host for nutrients and water. The parasitic seed germinates to form tubercle (rhizome) on host plant root in autumn and winter. Under suitable temperature and water conditions in March to early April, the tubercle begins to develop quickly, and emerges out of the ground as a low-growing inflorescence in late April to early May. After about 1 month, the tissue above ground becomes senile and forms numerous mature seeds upon pollination by flies, then drop and disperse in the desert sand. The rhizome underground, however, stays connected to the host root staying dormant for several years until the next development cycle begins. As an ethno-pharmacologically important and endangered medicinal species, the production of *C. songaricum* is far from meeting the demand, one tough problem is that the seed is very difficult to germinate due to the secretion of germination inhibitors such as abscisic acid and thicker wax coat which inhibits absorption of water and air that are essential for seed germination (Dong et al., 2010; Chen et al., 2011). For successful seed germination and development of *C. songaricum*, 5 years or even longer time is needed under natural conditions. Further, the studies on the mechanism of seed germination and development under natural parasitizing environment are largely undocumented to date. Compounding the matter further is the over-exploitation of stems and inflorescences (before seed maturation) for their popularity has proven detrimental to the preservation and conservation of *C. songaricum* on an ecological scale. Hence, for conservation of these medicinal plants a sustainable harvesting and manual cultivation technique is warranted (Cui et al., 2013).

Addressing this key issue, several tests were performed on improving seed germination rate (Dong et al., 2010). This includes breaking seed cover with ultrasonic treatment, soaking and then removing seed cover with a series of chemicals such as sodium hydroxide and potassium permanganate, or seed treatment with hormones such as gibberellins (GA) and naphthylacetic acid (NAA), or treatment with extracts of *N. tangutorum* root, and/or a combination of the above methods (Luo et al., 2013). But these methods exhibit challenges such as low germination rate, environment pollution, etc. Further, to date no one method has successfully been applied in sustainable production on a large scale. Other alternative methods yielding long-term reproducible solutions to sustainable production needs further investigation taking into consideration their development under natural parasitic habitats.

Endophytes are an integral part of plant biology and are perceived as host organs in and of themselves serving as important components of plant micro-ecosystems (Christian et al., 2016; Jia et al., 2016). Endophytes, especially fungal endophytes play important beneficial roles in host plant development and physiology including increased stress tolerance, enhanced root growth and provision for special nutrition and water (Wani et al., 2015). It is hypothesized that over long periods of co-evolution the endophytes not only establish special relationship with one another but can also significantly influence the formation and accumulation of certain key metabolic products altering the quality and quantity of crude drugs derived from medicinal plants (Faeth and Fagan, 2002; Firáková et al., 2007; Jia et al., 2016). Recently, a comprehensive review of published data on medicinal plants and endophyte relationships obtained in the last 30 years suggested that medicinal compounds could be effectively produced on a biotechnological scale if the relationships and the conditions that promote such production are clearly understood and cultivable endophytic fungi associated with medicinal plants be successfully established (Jia et al., 2016). The factors affecting the host-endophytic relationships and endophyte assembly in plant-parasitic interactions are limited, but previous results have suggested an important role for fungal endophytes and the diversity of fungal assemblages in key trophic interactions (Chen et al., 2005; Zhang et al., 2006; Behie et al., 2012).

Nearly 18 years ago, research showed no overlap of endophytic fungal assemblages of parasitic *Cuscuta reflexa* and it host *Cucurbita maxima* (Suryanarayanan et al., 2000). However, recent studies have highlighted the rich and diverse endophyte fungal communities in the hemi-parasitic plant *Macrosolen tricolor* and its host plant *Camellia oleifera*, and that they were always distributed in nearly the same orders of the four classes of Ascomycota and one class of Basidiomycota (Zhou et al., 2014). Surprisingly, the endophytic fungal assembly are shown to exhibit clear shifts between the parasitic plant and its host, for example, *Arceuthobium americanum* and its host *Pinus contorta* (Martin et al., 2012), and *Phoradendron perrottettii* and its host *Tapirira guianensis* (Teixeira-Costa and Ceccantini, 2016). In other studies, it has been demonstrated that endophytic fungi from parasitic *Viscum coloratum*, secrete cellulolytic enzymes which can degrade cellulose assisting the haustorium of mistletoe to break through the cell wall as well as intracellular tissue spaces within the host *Pterocarya stenoptera* (Ding et al., 2008). Besides the above roles of endophytes in parasitic development, they also exhibit profound influence on seed germination, and it is a well-established fact that *Mycena osmundicola* and *Mycena dendrobii* are essential for seed germination of *Gastrodia elata* by secreting indoleacetic acid (Xu and Guo, 1989; Guo and Wang, 2001). Recent investigations are also providing new and exciting information on the potential of cultivable endophytic fungi (associated with medicinal plants) as reliable sources of plant secondary metabolite accumulation and bioactivity (Nisa et al., 2015; Chagas et al., 2017; Cui et al., 2017; Ran et al., 2017). While the importance of endophytic fungi to the growth and development of host plants is increasingly being substantiated, exploiting these endophytic relationships for production of medicinal plant-based drugs and mechanistic understanding of the benefits of these exact relationships is still very limited.

To gain insights into the fungal endophytes and the diversity of fungal assembly in *C. songaricum* and its host *N. tangutorum*, here we (1) investigated the genetic relationship, community assembly and distribution characteristics of endophytic fungi across the different developmental stages of *C. songaricum* parasitizing *N. tangutorum*. (2) Evaluated the potential of the identified endophytic fungal assembly for functional interaction, providing evidence for the application of a target endophytic fungal spp. for promoting *C. songaricum* seed germination under *in vitro* axenic culture conditions. Our results demonstrate that parasitic interaction is a significant factor in shaping endophytic fungal assemblages. We provide evidence for the involvement of few endophytes in plant development and physiology, enabling promotion of seed germination of *C. songaricum*. The information obtained from this study provides new insights into the tripartite interactions of *N. tangutorum* hosts, root-parasitic plant *C. songaricum*, and its fungal endophytes. Our study highlights the potential for exploiting alternative bioengineering approaches to sustainable production of *C. songaricum*.

## Materials and Methods

### Plant Material Collection and Sample Preparation

**FIGURE 1:**
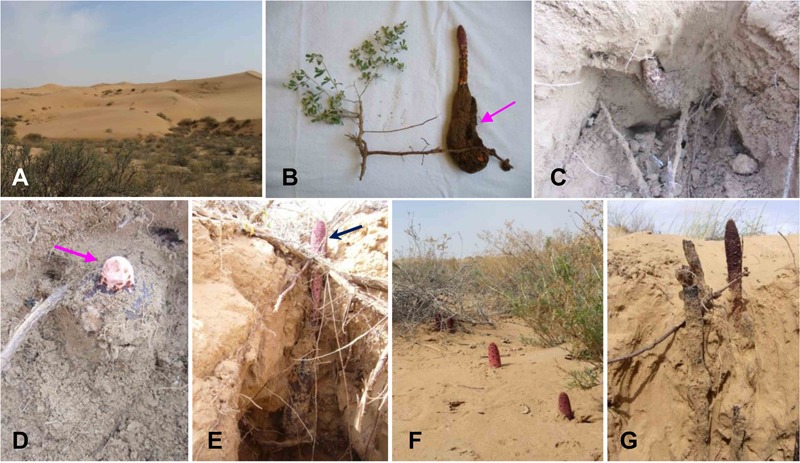
Habitat and root-parasitic development of *Cynomorium songaricum* on its host *Nitraria tangutorum*. **(A)** *C. songaricum* and its host *N. tangutorum* most often located in the peripheral regions of desert; **(B)** *C. songaricum* always parasitizes and connects with the root xylem and phloem of *N. tangutorum*; **(C)** A granulated rhizome is the living state of *C. songaricum* named the Tubercle (T) stage more so in the cold seasons; **(D)** The granulated rhizome will sprout when the temperature rises in March or April, named the Sprouting (S) stage; **(E)** *C. songaricum* emergence above ground, which will be collected as herb in April and May, this development is named the Unearthing (U) stage; **(F)** After about 1 month of growing out of ground, the mature seeds will be covered on the around dark-red head of *C. songaricum*, which is called the Maturing (M) stage; **(G)** After about 1 month, the stem and rhizome will decline and wither, named Atrophy (A) stage, and hence one development cycle is completed.

Healthy rhizomes of *C. songaricum* (CSR), parasitized root (SNT), and non-parasitized root (NT) of *N. tangutorum* were collected from the peripheral regions of Tengger Desert of China (38°44′–39°04′ N, 105°15′–105°22′E, ≈1305 m). (**Figures 1A,B**). For each plant species, only those samples with the same identified genotype (KX813699 for CSR, and KC813691 for SNT and NT) were used to conduct experiments in this study. Five developmental stages of *C. songaricum*, Tubercle (T), Sprouting (S), Unearthing (U), Maturing (M), and Atrophy (A), respectively, were sampled in November, March, May, June, and September from 2015 to 2016 (**Figures 1C–G**). The collected samples were cleaned with running water, surface-sterilized with 75% ethanol for 1 min, 5% NaOCl for 5 min, and finally washed twice with sterile distilled water. One part of these samples were cut to particles about 1 mm^3^, then placed in DNA-Be-Locked reagent (Sangon Biotech, Shanghai, China) to immobilize DNA for endophytic mycobiome study, while the other part was used for isolation of culturable endophytic fungi. Overall, eight plant samples were mixed and used in each treatment in CSR, SNT, and NT, respectively, and each treatment was set up as three independent replicates unless mentioned otherwise.

### Analyses of Fungal Assembly in *C. songaricum* and Its Host *N. tangutorum*

To investigate the distribution of fungal endophytes between *C. songaricum* and *N. tangutorum*, genomic DNA of surface-sterilized CSR, SNT, and NT was extracted with Plant DNA Mini Kit (Omega Bio-Tek, Doraville, GA, United States) following manufacturer’s instructions.

PCR amplification of the internal transcribed spacer region 2 (ITS2) in rRNA gene regions were performed using primer pairs ITS3F: GCATCGATGAAGAACGCAGC and ITS4: TCCTCCGCTTATTGATATGC. The PCR reaction system (25 μL) contained 1 μL of each primer (10 μM), 2 μL of template (50–100 μg/μL), 1 μL of dNTP (10 mM), 0.2 μL of TaqDNA Polymerase (5 U/μL), 2.5 μL of 10× buffer, 17.3 μL of ddH_2_O. PCR reactions were carried out as follows: 3 min of initial denaturation at 94°C, followed by 40 cycles of: 94°C for 30, 47°C for 30 s, and 72°C for 45 s; and final extension of 7 min at 72°C. PCR products were recovered from agarose gels using PureLink Quick gel extraction Kit (Thermo Fisher Scientific, United States) according to the manufacturer’s instructions. After quantification of DNA concentration using Nanodrop ND-1000 with software *ver.*3.3 (Thermo Scientific, United States), all PCR products were sent for high-throughput amplicon sequencing (in triplicates) using the Illumina Miseq platform (United States) at the Services Facility (Sangon Biotech, Shanghai, China).

### Analyses of Mycobiome Dynamics

To shed light on the community dynamics of endophytic fungi, the rhizome samples from five different development stages, i.e., T, S, U, M, and A stage of *C. songaricum* were collected, surface sterilized, DNA extracted, PCR amplified, and high-throughput MiSeq analyzed. The community composition changes of endophytic fungi based on their developmental structures were analyzed and further compared.

### Bioinformatic and Statistical Analyses

Raw data of pyrosequences were analyzed using the Quantitative Insights into Microbial Ecology (QIIME) pipeline 1.8.0 (Caporaso et al., 2010). MOTHUR software was adopted to analyze retrieved sequences (Schloss et al., 2009). Paired-end sequences were assembled with software FLASH *ver*1.2.3 (Magoč and Salzberg, 2011). Those sequences that had low quality (multiple N, base pair mismatch, chimeras, etc.) or length shorter than 50 bp were omitted. Sequences were then denoised according to the method of Reeder and Knight (2010). The sequences with high quality were clustered into operational taxonomic units (OTUs) at a 97% similarity threshold using the USEARCH algorithm (Edgar et al., 2011). A “sub.sample” command was performed and all samples were adjusted, by random selection, to the sample with the lowest number of sequences (20,000–65,000 sequences per sample for Illumina and ion torrent data, respectively). Taxonomy was classified to fungal OTUs using the RDP option in the parallel_assign_taxonmy_rdp.pyfunction with mini-confidence of 0.8 (Wang et al., 2007). The reference OTUs were obtained from the UNITE database (Version 7^2^). The difference in fungal communities of all samples was by ANOVA statistics, Multi_Dimension and VennDiagram package in R software based on OTUs. The alpha diversity was analyzed through diversity indices including Chao-1 richness index predicting the total numbers of OTUs in each sample, Shannon, Simpson index and coverage representing coverage rate. Beta diversity was exhibited through Principal Component Analysis (PCA), Principal Co_ordinates (PCoA), non_multi_dimension_analysis (NMDS), Unweighted pair group method with arithmetic mean (UPGMA) and Unweighted uniFrac. The genetic relationship of fungal OTUs and correlation was analyzed by Hierarchical Clustering dendrogram, Bray_Crutis_treeand“OUT_co_network”, based on R software.

### Isolation and Identification of Culturable Endophytes

To isolate and culture endophytic fungi, surface-sterilized samples were cut to 2–3 mm cubes, placed on potato dextrose agar (PDA) at 25°C in the dark. A total of 80 cubes were randomly selected to place on 20 PDA plates, in each plate were placed four tissues from CSR, SNT and NT samples, respectively. Three plates without cutting were used as culture-controls. The hyphae were sub-cultured multiple times to obtain pure cultures, then were recorded, numbered, and deposited in the Institute of Applied Chemistry, Shanxi University.

Identification of fungus was performed by methods described in Cui et al. (2015). Briefly, ITS rDNA region was amplified using ITS1 (5′-TCCGATGGTGAACCTGCGG-3′) and ITS4 (5′-TCCTCCGCTTATTGATATGC-3′) primers. The PCR reactions were performed as described earlier (Cui et al., 2015). The PCR products were sequenced at Sangon Biotech (Shanghai) Co., Ltd., aligned and blast analyzed against the NCBI database^1^. The isolates were assigned to different OTUs at a 97% similarity threshold of ITS sequences. Indicator genus or species were identified through comparing the similarity sequence with the threshold of 97 and 99%. Isolation rate (IR) = x/y × 100% (x, number of fungal isolations; y, number of samples per isolation).

### Analysis of Fungal Activity Promoting Seed Germination

All the culturable fungi in CSR and SNT were investigated to detect their activity for promoting seed germination of *C. songaricum in vitro*. The fungal cultures were inoculated into 500 mL Erlenmeyer flask containing 200 mL of liquid PDA medium. Shake flasks were incubated at 220 rpm on rotary shaker at 25°C for 7 days. Cultures were then separated into mycelial mat and filtrate using Whatman No. 1 filter paper. The mycelial mat was ground, weighed, and water added (1/10 mg/mL), after homogenization the mycelial extract was stored at 4°C until further use. Both filtrate and mycelial extract were sprayed on Whatman GF/A glass-fiber filter, respectively, and placed on petri dishes and sealed to keep moisture. *C. songaricum* seeds were treated to remove seed coat by soaking in 1 mol/L NaOH for 5 h, followed by (3×) rinsing with ddH_2_O, placed on moist filter paper, and incubated in the dark at 25°C. Care was taken to use appropriate negative controls in each case. Each group had twenty such repetitions for statistical analysis. The germination time and rate were observed and recorded every day for statistical analysis.

### Sequence Accessions

Sequence data from 281 representative genera or species of OTUs from culturable endophytic fungi were deposited in GenBank under accession numbers KY379536-KY379816. ITS2 sequences that were identified in an earlier study are deposited under the GenBank accession numbers KX813698-KX813705 (*C. songaricum*) and KC813691-KC813696 (*N. tangutorum*). Sequences obtained from next-generation sequencing were deposited in the Sequence Read Archive (SRA) database in NCBI^2^ (SRA Accession: SRP128939).

## Results

### Diversity of Fungal Assemblages in *C. songaricum* and Its Host *N. tangutorum*

**FIGURE 2:**
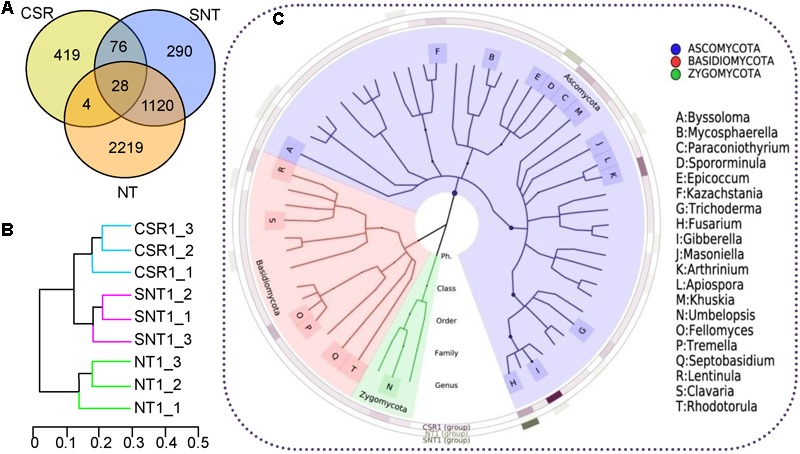
Distribution of endophytic fungi among *C. songaricum* (CSR), parasitized *N. tangutorum* (SNT), and non-parasitized *N. tangutorum* (NT) based on high-throughput sequencing of ITS2 rRNA gene. The fungal community composition and phylogenetic relationship were determined based on operational taxonomic unit (OTU) using a 97% similarity threshold. **(A)** Venn diagram of OTUs among CSR, SNT, and NT. **(B)** Bray tree plot showed relationship of fungal composition in CSR is closer to SNT than that of CSR to NT. **(C)** Phylogenetic tree of the top 100 genera, the most dominant twenty genera were marked, and the outer cycles were heatmap in three samples which showed that the fungi were orderly affiliated in Ascomycota, Basidiomycota, and Zygomycota.

To investigate the diversity of endophytic fungi the in root-parasitic plant *C. songaricum* (CSR), and to analyze the difference in fungal endophyte diversity among *C. songaricum*, parasitized and non-parasitized *N. tangutorum*, SNT and NT, respectively, the fungal communities in the three host plant types were assayed in the surface sterilized tubercle (T) stage (was a granulated rhizome of living state of *C. songaricum* in cold season), which was observed as the initial period of parasitization (**Figure 1C**). Through Illumina MiSeq analysis we obtained 309,764 high-quality reads covering 5,358 fungal OTUs. The number of reads per sample was 64,792, 84,447, and 160,525 covering CSR, SNT, and NT, respectively, of which they covered 527, 1,514 and 3,371 fungal OTUs, respectively (**Figure 2A**). There were a total of 28 commonly shared fungal OTUs found in all three hosts, while the commonly shared fungal OTUs between NT and SNT, SNT and CSR, and CSR and NT were 1,120, 76, and 4, respectively. According to Venn distribution analysis based on ITS2 ribosomal rRNA genes, the similarity was among the three host plants was found to be 23.47, 3.88, and 0.10%, respectively (**Figure 2A**). Hierarchical Clustering dendrogram showed that fungal community of CSR had close genetic relationship to SNT than that of CSR to NT (**Figure 2B**), which was also supported by principal co-ordinates analysis (Supplementary Figure S1).

A great number of observed endophytic fungi, exhibited fairly rich fungal diversity in these specialized desert plants. Analysis of species richness based on the comparison of many alpha diversity (identification of all species present) indexes such as; richness number, Shannon index, ACE richness estimator, Chao_1 index, and Simpson index among the three host plant types suggested successively increasing species richness among CSR, SNT, and NT, respectively (**Table 1**). Except for the identification of certain unclassified species, most of the endophytic fungi belonged to the phylum Ascomycota, followed by Basidiomycota and Zygomycota. A well-known xerophilic fungus identified among them was *Aspergillus penicillioides*. In other classified species, the most abundant fungal genus was *Gibberella* (5.92%) comprising three species *G. tricincta, G. zeae*, and *G. fujikuroi*. While, the most abundant of the *Gibberella* species was the *G. tricincta* which covered 10 sub-species in CSR, but were not found in both SNT and NT. The next abundant genus that was identified was the *Fusarium* spp. comprising 0.31% in CSR, 0.67% in SNT (the most abundant genus), whereas it could not be identified in NT sample types. To better understand the diversity and distribution of fungal endophyte genera among the three host plant types a phylogenetic analysis of relationships was constructed from the top 100 known genera in **Figure 2C**. Taken together, the results showed that the three host plant types had different specificities for fungal colonization.

**Table 1:** Comparison of alpha diversity indexes among *Cynomorium songaricum*, parasitized and non-parasitized *Nitraria tangutorum*, respectively.

Sample	Richness	Shannon	ACE	Chao1_index	Cover.	Simpson
CSR1_1	64,792	1.29 ± 0.43	407 ± 50.70	380.2 ± 42.3	1.0	0.63 ± 0.14
SNT1_1	84,447	2.22 ± 0.06	1864.29 ± 93.0	1248.89 ± 31.52	0.99	0.27 ± 0.01
NT1_1	160,525	2.81 ± 0.08	3619.97 ± 420.2	3040.69 ± 22.81	0.98	0.35 ± 0.03

*Richness, observed number of operational taxonomic units (OTUs); Shannon, Shannon index estimator; ACE, ACE richness estimator; Chao1_index, Chao1 richness estimator; Cover., Good’s coverage estimator; Simpson, Simpson index estimator. Values represent Mean ± SD (n = 3). The rarefaction of alpha diversity calculation included 309,764 sequences across nine samples.*

### Fungal Community Dynamics Across the Developmental Stages of *C. songaricum* Parasitizing *N. tangutorum*

**FIGURE 3:**
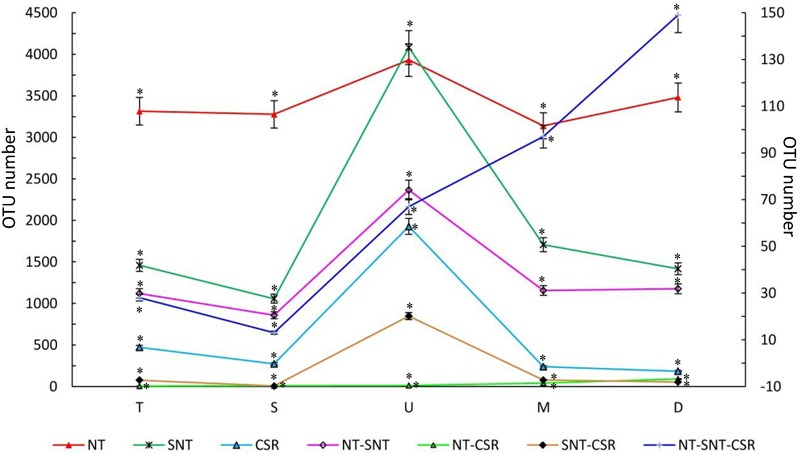
Dynamics of endophytic fungal community in *C. songaricum* (CSR) across five developmental stages of parasitized *N. tangutorum* (SNT) and non-parasitized *N. tangutorum* (NT). NT-SNT, NT-CSR, SNT-CSR, and NT-SNT-CSR represented the shared OTUs between or among of NT, SNT, and CSR, respectively; T, S, U, M, and A represented five developmental stages of Tubercle, Sprouting, Unearthing, Maturing and Atrophy, respectively. *Y*-axis shows value of OTU number, and left *Y*-axis exhibits the OTU numbers of NT, SNT, CSR, and NT-SNT and right *Y*-axis indicates the data of NT-CSR, SNT-CSR, and NT-SNT-CSR. Results are presented as the means ± SD of three replicates. ^∗^*P* < 0.05, ^∗∗^*P* < 0.05, the difference in fungal communities of all samples was by ANOVA statistics.

Next, we aimed to characterize the dynamics of fungal community composition in each successive developmental cycle of *C. songaricum* parasitizing *N. tangutorum* (**Figure 1**). For this we analyzed the five developmental stages starting from T to A (**Figure 1C** was the T stage which exhibited initial parasitic development stage of *C. songaricum* with a living stage of granulated rhizome parasitizing root of *N. tangutorum* in cold season, and the granulated rhizome sprout when the temperature rises in March or April, named S stage as **Figure 1D**. Then the next development was U stage when *C. songaricum* grows out of ground in April or May as **Figure 1E**. After about 1 month of growing out of ground, the mature seeds will be covered on the around dark-red head of *C. songaricum*, which called M stage as **Figure 1F**, and after June, the stem and rhizome decline and wither, was A stage in **Figure 1G**) i.e., development stages depicting rising temperature in the desert conditions from November to May/June. The fungal OTU analysis suggested an increase in OTUs among CSR, SNT, and NT up until the U stage, while, the fungal OTUs decreased from stages U to A, probably due to the selective action of host plants (**Figure 3**). Interestingly, however, the fungal OTUs in NT, SNT, and CSR reached a maximum number at U stage of *C. songaricum*, respectively, which suggested that endophytic fungi may have had an elevated growth activity when root-parasitic plant grows out of ground/emerges above ground (**Figure 3**). In contrast, the number of shared OTUs among NT-SNT-CSR, increased successively from 28 to 202, from S to A stage, while the number of shared OTUs in NT-CSR and SNT-CSR stayed constant and/or slightly, respectively (**Figure 3**).

**FIGURE 4:**
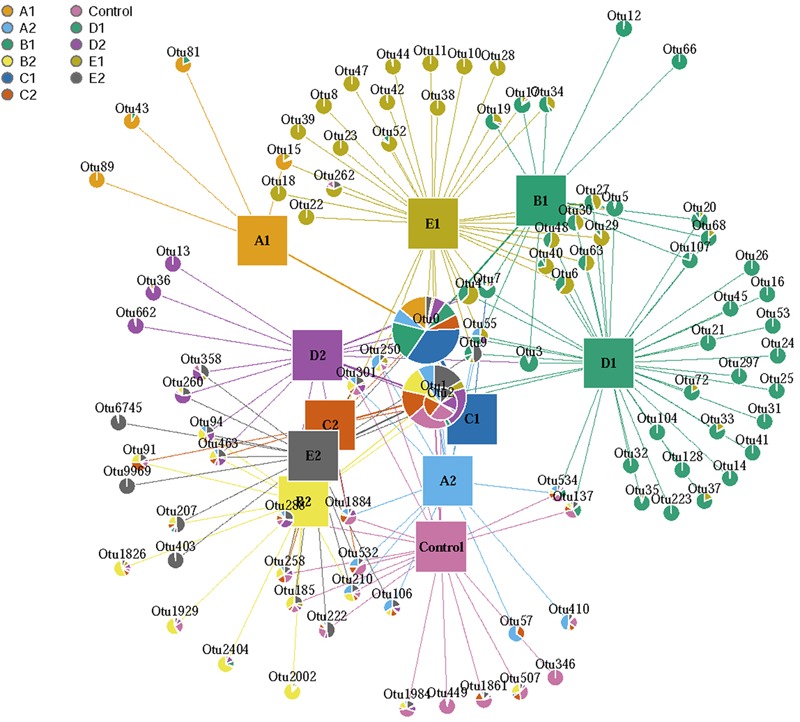
Network map based on the first 100 OTUs in *C. songaricum* (CSR), parasitized *N. tangutorum* (SNT), and non-parasitized *N. tangutorum* (NT) based on ITS2 region. A1, B1, C1, D1, and E1 represented CSR from five developmental stages: Tubercle, Sprouting, Unearthing, Maturing, and Atrophy stage; A2, B2, C2, D2, and E2 represent SNT samples corresponding to the five stages of CSR development, respectively; Control, represents NT in hypothetical T stage. The data was analyzed by Quantitative Insights into Microbial Ecology (QIIME) and the network was drawn using the graph package in R software.

To understand further the relationship between the shared OTUs in the five developmental stages of *C. songaricum* growth, a co-occurrence network (pattern) analysis was conducted using the first 100 OTUs. Network analysis indicated a close association of fungal communities in the three host plant types with respect to their developmental stages, i.e., T to A stage of *C. songaricum* growth (**Figure 4**). Further, aiming to understand their genetic relatedness, the first 50 representative fungal OTUs were used for phylogenetic analysis (Supplementary Figure S2). This tree indicated that in the top 20 successfully classified OTUs (apart from the unclassified species), most of them belonged to well-known identified fungal spp. including *Candida neorugosa* (OTU3), *Lentinula edodes* (OTU5), *Devriesia lagerstroemiae* (OTU11), *Gibberella tricincta* (OTU15), *G. fujikuroi* (OTU16), *Fusarium solani* (OTU17), *Tropicoporus linteus* (OTU18), and *Aspergillus sydowii* (OTU20). These OTUs were found to frequently associate with CSR during its development, and made up their composition comprising of unique communities (**Figure 4** and Supplementary Figure S2). Interestingly, five out of these eight OTUs showed a clear association with the developmental stages of S and M (**Figure 4**). Overall, the fungal diversity (OTU enrichment) observed in the developmental stages of CSR and SNT, respectively (**Figure 4**) were distinct in nature and little to no overlap was observed among the identified fungal OTUs.

### Fungal Endophytes Recovered as Seed Germination Promotors

A total of 281 fungal endophytes were isolated and cultured from surface-sterilized CSR, SNT, and NT, respectively, comprising the five different developmental stages of T to A (Supplementary Tables S1, S2). Results were classified into at least 31 genera, with their average *IR* being 31.9%. The most abundant fungus to be isolated was *Fusarium* spp. followed by its teleomorph *Gibberella* spp., *Penicillium* spp., *Ilyonectria* spp., *Aspergillus* spp., and *Alternaria* spp., respectively (Supplementary Tables S1, S2). The fungal dynamics that were observed in isolation experiments from the five developmental stages were consistent with those of the results obtained from high throughput sequencing (**Figure 2C** and **Table 2**).

**Table 2:** Endophytic fungi with bio-activity for promotion of *C. songaricum* seed germination.

Isolate origin	Classification	GenBank No.	Closest match (GenBank No.) – % seq identity	Extract	Seed germination rate effected by fungus
CSR in Sprouting stage	*Fusarium* sp.	KY379544	*F. redolens* (KU901531.1) – 100%	Ferm. broth	45.00%
SNT in Sprouting stage	*Fusarium* sp.	KY379572	*F. nematophilum* (KU9324800.1) *–* 100%	Ferm. broth	25.00%
CSR in Maturing stage	*Aspergillus* sp.	KY379684	*A. terreus* (KY425727.1) – 100%	Ferm. broth	0.50%
SNT in Maturing stage	*Clonostachys* sp.	KY379716	*C. rosea* (LT576164.1) – 100%	Ferm. broth	0.50%
SNT in Atrophy stage	*Fusarium* sp.	KY379764	*F. nematophilum* (KX272852.1) – 100%	Ferm. broth	15.00%

*CSR, sample of rhizome from C. songaricum; SNT, parasitized Nitraria tangutorum; Ferm. broth, extract of fungal fermentation broth.*

To examine the effect of different endophytic isolates on the growth and development of parasitic *C. songaricum*, fermentation broth and mycelial extract of each isolated and culturable fungus was tested for promotion of *C. songaricum* seed germination *in vitro* (Supplementary Figure S3). Results showed that the fermentation broth from the five isolates comprising the species of *Fusarium redolens, Fusarium nematophilum, Aspergillus terreus*, and *Clonostachys rosea* exhibited seed germination promotion activity in *C. songaricum*. As shown in **Table 2** we observed that *F. redolens* (KY379544, from CSR in Sprouting stage) and *F. nematophilum* (KY379572, from SNT in Sprouting stage) showed the greatest frequency of germination efficiency, i.e., 45 and 25%, respectively (Supplementary Figure S3). The results showed that endophytic fungi participated in the root-parasitic interaction of *C. songaricum* with *N. tangutorum*, presenting evidence of their potential as target endophytic fungal spp. for seed germination enhancement and a probable strategy for conservation and cultivation of the endangered *Cynomorium* medicinal plant species.

## Discussion

With the rapid development of high-throughput sequencing methods and bioinformatics tools, a huge number of cryptic fungi are being discovered (Yang et al., 2016). Previous estimates based on high-throughput sequencing suggest as many as 5.1 million fungal species and a significant proportion of which is estimated to be found in plant–endophyte relations (Blackwell, 2011). Recently, the use of “meta-omic” technologies such as metagenomics has helped to realize the potential of endophytes in plant-fungal interaction studies (Kaul et al., 2016). While many aspects of endophytic biology are still unknown, metagenomic approaches in identifying sequences of interacting partners is unraveling the nuances of plant–endophyte relationship. The identified sequences help exploring endophytic species distribution, community structure, lifestyles and nutrition modes, and provide objective insights into endophytic fungal richness, ecological function and fungal-mediated processes in host plant (Ottosson et al., 2015). While, an urgency to establish sustainable programs for conservation and resource utilization of medicinal plants has long been proposed (Cui et al., 2013), further studies on mechanisms and exploitation of co-existing endophytes is still largely limited. Here, in our study we aimed at understanding the diversity of fungal endophytes in one such ethnopharmacological relevant spp. of the genus *Cynomorium*, that forms root-parasitic associations with *N. tangutorum*. Previously, a pioneering study on characterization of endophytic fungal communities in 29 traditional Chinese medicinal plants had suggested a predominant occurrence of fungal endophyte species of the taxa; *Alternaria, Colletotrichum, Phoma, Phomopsis, Xylariales*, and mycelia sterilia (Huang et al., 2008). Further, quite interestingly to date most other fungal endophytes discovered have been shown to be the fungal species that could be obtained easily and isolated commonly in the environment, such as *Gibberella* spp., *Fusarium* spp., *Aspergillus* spp., *Candida* spp., and *Penicillium* spp. (Strobel and Daisy, 2003). In this study, using high-throughput sequencing we detected dominant genera/order of endophytic fungi at different distribution levels among our CSR, SNT, and NT samples (**Figures 1, 2A,C** and Supplementary Figure S2). Our results concur with previously reported findings that under traditional methods used in laboratory conditions only 0.1–1.0% of endophytic fungi could be obtained because of artificial selection pressure (Amann et al., 1995). However, considering the medicinal value of *Cynomorium* spp. in the clinic as well as in folk-medicines information on the endophytes found in the root-parasitic interactions of *C. songaricum* and its host *N. tangutorum* could be deemed invaluable.

Plant endophytic fungi are shown to originate mainly from seeds through vertical transmission or from the environment through horizontal transmission (Christian et al., 2016; Guo et al., 2017). However, studies on endophytic fungal distribution in parasitic plants and its host are more complicated, for fungal colonization and movement within the two plants can either be facilitated via xylem tubes (Compant et al., 2010) or through apoplasts, intercellular space (Maheshwari et al., 2013). For example, it was found that the mistletoe *Phoradendron perrottettii* and its host tree *Tapirira guianensis* shared some common endophyte species while still maintaining distinct communities (de Abreu et al., 2010). Similarly, in our studies (**Figure 2A**) we found that CSR and its host SNT shared much more OTUs (76 in total) for endophytic fungi, compared to CSR and NT (4 in total). The reason for this observed discrepancy may be that *C. songaricum* is succulent while *N. tangutorum* is a ligneous plant, which may lead to the damage of *C. songaricum* tissue by sand more easily as compared to *N. tangutorum*. Thus, it can be hypothesized that the fungal invasion and colonization to *C. songaricum* is easier than *N. tangutorum.* This may probably explain the observed differences in the number of unique fungi in CSR (419) being more than those in SNT (290) (**Figures 2A,C**). Further, we also found that NT and SNT together shared the maximum numbers (1120) of endophytic fungi, which we believe supports the notion of species specificity and selective specificity existing between endophytes and their hosts (Rai and Agarkar, 2016). On the other hand, the parasitic connection in CSR and SNT would lead to chemical exchange, and further influence selection and/or fungal inhibition. Thus, the genetic distance of fungal communities from SNT intermediated between CSR and NT, however, results suggest that the fungal assemblages are more associative toward CSR than NT (**Figure 2B**).

In one life cycle, *C. songaricum* grows originating from a tubercle (about several millimeters) to an exuberant organism (about 0.5 m) spanning the T to A developmental stage, then they shrink gradually toward the end of the year. With the growth of root tips or other tissue in sand, more and more environmental fungi would invade and colonize plants via the tissue that is injured by sand particles. Thus, it can be speculated that the amount of endophytic fungi increases from T to U developmental stage with the rising desert temperatures (**Figure 3**). After the peak (U stage), the fungal number decreased until to A stage (Kruh et al., 2017). The invaded fungi may be subjected to selection pressure from the host plants (Nikolov et al., 2014; Rai and Agarkar, 2016), and only part of them capable of adapting to the host internal environment, may survive and multiply in the host plants (Huang et al., 2017). Hence, the longer the plants grow, the more is the shared endophytic fungi among CSR, SNT, and NT (**Figure 3**). On the other hand, because of exchange of fungi through parasitic connections (Kruh et al., 2017), the shared fungi between CSR and SNT were more than that between CSR and NT in each developmental stage. This suggests a most probable co-evolution and natural selection among host species (Ambrose et al., 2014).

The role and mechanism of endophytes in parasitic interaction between *C. songaricum* and *N. tangutorum* remains largely uninvestigated, and our results provide key cues that could further be used for functional evaluation of fungal assemblages in isolation or in unison. Previous evidence indicates that products from endophytic fungi affect physiology and development of host plants (Rodriguez and Redman, 2008). For example, strigolactones produced almost exclusively from parasitized host plant, serve as essential signal molecules stimulating the germination of parasitic plant. Recent evidence that strigolactone biosynthesis can be regulated by gibberellins which were produced by endophytic *Gibberella* spp. and *Fusarium* spp. provides further opportunities for analyzing the roles of endophytic fungal assemblages (Lale and Gadre, 2010; Troncoso et al., 2010; Ito et al., 2017). Interestingly, even in our study both *Gibberella* spp. and *Fusarium* spp. were identified as the dominant fungi in parasitic *C. songaricum* and its host *N. tangutorum*. And, further analysis of relevance and function of the *Fusarium* spp. on the growth and development of *C. songaricum* was tested using *in vitro* axenic conditions, which suggested that the *Fusarium redolens* (KY379544) can serve as a target endophytic fungal species in promoting *C. songaricum* seed germination (Supplementary Figure S3). However, it remains to be investigated whether they are involved in the tripartite interaction of fungal endophytes with the root-parasitic plant and/or its host. While, some endophytic bacteria were also shown to possess the ability of promoting host seed germination (Shittu et al., 2009; Xu et al., 2014), studies with respect to the use of fungal endophytes as sources of medicinal host similar bioactive component (biosimilar) is only now beginning to be addressed to its full potential (Jia et al., 2016). In this study, five isolates (*F. redolens* KY379544, *F. nematophilum* KY379572, *A. terreus* KY379684, *C. rosea* KY379716, and *F. nematophilum* KY379764) showed the ability of promoting seed germination, which suggests that endophytic fungi may play important roles in root-parasitic plant interactions and parasitic host growth and development.

## Conclusion

This study firstly demonstrates that parasitic behavior of *C. songaricum* on *N. tangutorum* could significantly affect the inhabiting distribution, growth and decline, and other plant fungal endophyte dynamics. We show that the endophytic fungal community shifts between the parasitic plant and its host, which helps one to understand the significance of such ecological relationships of parasite-endophyte-host organism(s) in tripartite associations. Some of the endophytes we isolated in this study exhibit the ability to promote *C. songaricum* seed germination, which concurs with previously suggested studies that endophytes could significantly influence the production and accumulation of medicinal host metabolites. Our results in the ethnopharmacological relevant *Cynomorium* spp., highlights the important and integral part fungal endophytes occupy in the *C. songaricum’*s desert ecosystem, thus playing a vital role in parasitic development and physiology. Our results provide evidence for future investigations on novel ways for improving the parasitic survival rate and production of *C. songaricum*, which in the long-run may aid maintenance and sustainable development of desert plant ecology.

## Author Contributions

J-LC and GZ designed the research, performed the experiments, and analyzed the data. J-LC collected the samples. J-LC, VV, and GZ co-wrote the paper.

## Conflict of Interest Statement

The authors declare that the research was conducted in the absence of any commercial or financial relationships that could be construed as a potential conflict of interest.

## Footnotes

1 http://www.ncbi.nlm.nih.gov

2 https://submit.ncbi.nlm.nih.gov
