# Supplementary material for: Partitioning of Fungal Endophyte Assemblages in Root-Parasitic Plant Cynomorium songaricum and Its Host Nitraria tangutorum
Source: Front Microbiol. 2018 Apr 5;9:666. doi: 10.3389/fmicb.2018.00666 (PMC5900785; doi:10.3389/fmicb.2018.00666)
Supplement: Supplementary file 1 [file Table_1.DOCX]

Supplementary Material

**Partitioning of Fungal** **Endophyte Assemblages in Root-parasitic Plant *Cynomorium songaricum* and Its Host *Nitraria tangutorum***

**Jin-Long Cui^1*^, Vinod Vijayakumar^3^, Gang Zhang^2*^**

^1^Institute of Applied Chemistry, Shanxi University, Taiyuan, China.

^2^[College](javascript:void(0);) [of](javascript:void(0);) [Pharmacy](javascript:void(0);), Shaanxi University of Chinese Medicine, Xianyang, China.

^3^College of Food, Agricultural and Environmental Sciences, Department of Food Science and Technology, The Ohio State University, Columbus, OH, USA

**^*^Correspondence:**

Jinlong Cui (cjl717@sxu.edu.cn) OR Gang Zhang ([jay_gumling2003@aliyun.com](mailto:jay_gumling2003@aliyun.com))

# Supplementary Tables

**1.1 Supplementary Table S1 Classification and distribution of endophytic fungi from *Cynomorium songaricum***

| **Code** | **Classification** | **Isolate number** | | | | | **Total** |
| --- | --- | --- | --- | --- | --- | --- | --- |
|  |  | **CSR-T** | **CSR-S** | **CSR-U** | **CSR-M** | **CSR-A** |  |
| 1 | *Acremonium* sp. | 1 | - | - | - | - | 1 |
| 2 | *Alternaria* spp. | - | - | 2 | - | - | 2 |
| 3 | *Aspergillus* spp. | - | 2 | 5 | 4 | 1 | 12 |
| 4 | *Clonostachys* spp. | 1 | - | 1 | - | - | 2 |
| 5 | *Dactylonectria* sp. | - | - | - | - | 1 | 1 |
| 6 | *Emericella* sp. | - | - | - | 1 | - | 1 |
| 7 | *Fusarium* spp. | 11 | 2 | 6 | 4 | 6 | 29 |
| 8 | Hypocreales spp. | - | 1 | - | 1 | - | 2 |
| 9 | *Ilyonectria* spp. | 3 | 2 | 4 | 3 | 6 | 18 |
| 10 | *Microascus* spp. | - | - | 5 | 2 | - | 7 |
| 11 | *Penicillium* spp. | 2 | 1 | 8 | 7 | 2 | 20 |
| 12 | *Peniophora* sp. | - | - | - | 1 | - | 1 |
| 13 | *Phanerochaete* sp. | - | - | 1 | - | - | 1 |
| 14 | *Rhizoctonia* sp. | - | - | 1 | - | - | 1 |
| 15 | *Talaromyces* sp. | - | - | - | - | 1 | 1 |
| 16 | *Trichurus* spp. | 3 | 1 | 1 | - | 6 | 11 |
| 17 | unidentified fungus | - | 1 | - | - | - | 1 |
|  | total | 21 | 10 | 34 | 23 | 23 | 111 |

*-, no isolated fungi; CSR-T, CSR-S, CSR-U, CSR-M and CSR-A, the rhizomes of C. songaricum at Tubercle, Sprouting, Unearthing, Maturing and Atrophy developmental stage, respectively.*

**1.2 Supplementary Table S2 Classification and distribution of endophytic fungi isolated from parasitized and non-parasitized *N. tangutorum***

| **Code** | **Classification** | **Isolate number** | | | | | | **Total** |
| --- | --- | --- | --- | --- | --- | --- | --- | --- |
|  |  | **SNT-T** | **SNT-S** | **SNT-U** | **SNT-M** | **SNT-A** | **NT(CK)** |  |
| 1 | *Alternaria* spp. | - | - | - | 9 | 5 | - | 14 |
| 2 | *Aspergillus* spp. | 1 | 2 | 1 | 2 | 2 | 6 | 14 |
| 3 | *Camarosporium* sp. | - | - | - | 1 | - | - | 1 |
| 4 | *Chaetomium* sp. | - | 1 | - | - | - | 3 | 4 |
| 5 | *Clonostachys* spp. | 2 | 2 | - | 1 | - | - | 5 |
| 6 | *Dactylonectria* sp. | - | - | - | - | 1 | - | 1 |
| 7 | *Emericellopsis* sp. | 1 | - | - | - | - | 1 | 2 |
| 8 | *Fusarium* spp. | 12 | 7 | 10 | 15 | 3 | 9 | 58 |
| 9 | Hypocreales sp. | - | - | - | 1 | - | - | 1 |
| 10 | *Ilyonectria* spp. | 1 | 1 | 4 | 4 | 3 | 3 | 16 |
| 11 | *Microascus* spp. | 1 | - | 1 | - | - | 2 | 2 |
| 12 | *Penicillium* spp. | 2 | 4 | 1 | 5 | 2 | 2 | 16 |
| 13 | *Preussia* sp. | - | - | - | - | 1 | - | 1 |
| 14 | *Purpureocillium* sp. | 1 | - | - | - | - | 1 | 2 |
| 15 | *Rhizopycnis* spp. | - | - | 3 | - | - | - | 3 |
| 16 | Sordariales spp. | - | 3 | - | - | - | - | 3 |
| 17 | *Talaromyces* sp. | - | 1 | - | - | - | - | 1 |
| 18 | *Trichothecium* spp. | - | - | - | 1 | 1 | - | 2 |
| 19 | *Trichurus* spp. | - | - | - | - | 2 | - | 2 |
| 20 | *Ulocladium* spp. | - | 1 | - | 1 | - | - | 2 |
| 21 | *Acrophialophora* sp. | - | - | - | - | - | 1 | 7 |
| 22 | Pleosporales sp. | - | - | - | - | - | 1 | 1 |
| 23 | Sporormiaceae spp. | - | - | - | - | - | 2 | 2 |
| 24 | *Thielavia* spp. | - | - | - | - | - | 2 | 2 |
| 25 | *Xylogone* sp. | - | - | - | - | - | 1 | 1 |
| 26 | unidentified fungus | 1 | 2 | - | - | 4 | 4 | 4 |
|  | Total | 24 | 27 | 24 | 44 | 29 | 40 | 170 |

*-, no isolated fungi; SNT-T, SNT-S, SNT-U, SNT-M and SNT-A, the root materials of parasitized N. tangutorum at Tubercle, Sprouting, Unearthing, Maturing and Atrophy developmental stage, respectively; NT, non-parasitized root of N. tangutorum.*
