# Supplementary material for: Partitioning of Fungal Endophyte Assemblages in Root-Parasitic Plant Cynomorium songaricum and Its Host Nitraria tangutorum
Source: Front Microbiol. 2018 Apr 5;9:666. doi: 10.3389/fmicb.2018.00666 (PMC5900785; doi:10.3389/fmicb.2018.00666)
Supplement: Supplementary file 2 [file Image_1.PDF]

frontiers

Supplementary Material

Partitioning of Fungal Endophyte Assemblages in Root-parasitic Plant *Cynomorium songaricum* and Its Host *Nitraria tangutorum*

Jin-Long Cui[1*], Vinod Vijayakumar[3], Gang Zhang[2*]

[1]Institute of Applied Chemistry, Shanxi University, Taiyuan, China.

[2]College of Pharmacy, Shaanxi University of Chinese Medicine, Xianyang, China.

[3]College of Food, Agricultural and Environmental Sciences, Department of Food Science and Technology, The Ohio State University, Columbus, OH, USA

[*]Correspondence:

Jinlong Cui (cjl717@sxu.edu.cn) OR Gang Zhang (jay_gumling2003@aliyun.com)

1 Supplementary Figures (A total of 3)

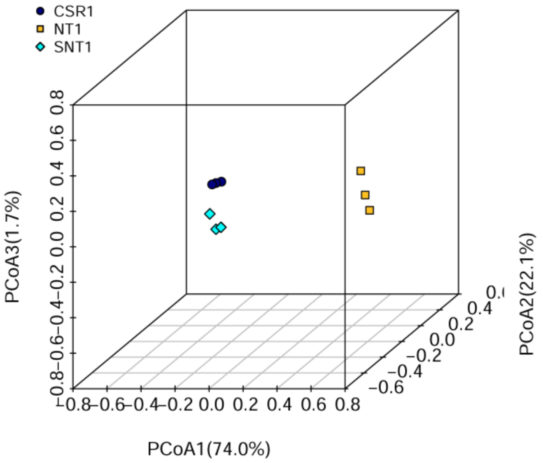

SUPPLEMENTARY FIGURE S1 Principal co-ordinates analysis (PCoA) of endophytic fungi among *C. songaricum* (CSR), parasized *N. tangutorum* (SNT) and non-parasitized *N. tangutorum* (NT) based on high-throughput sequencing of ITS2 rRNA gene. The relationship of fungal community of CSR is closer to SNT than that of CSR to NT.

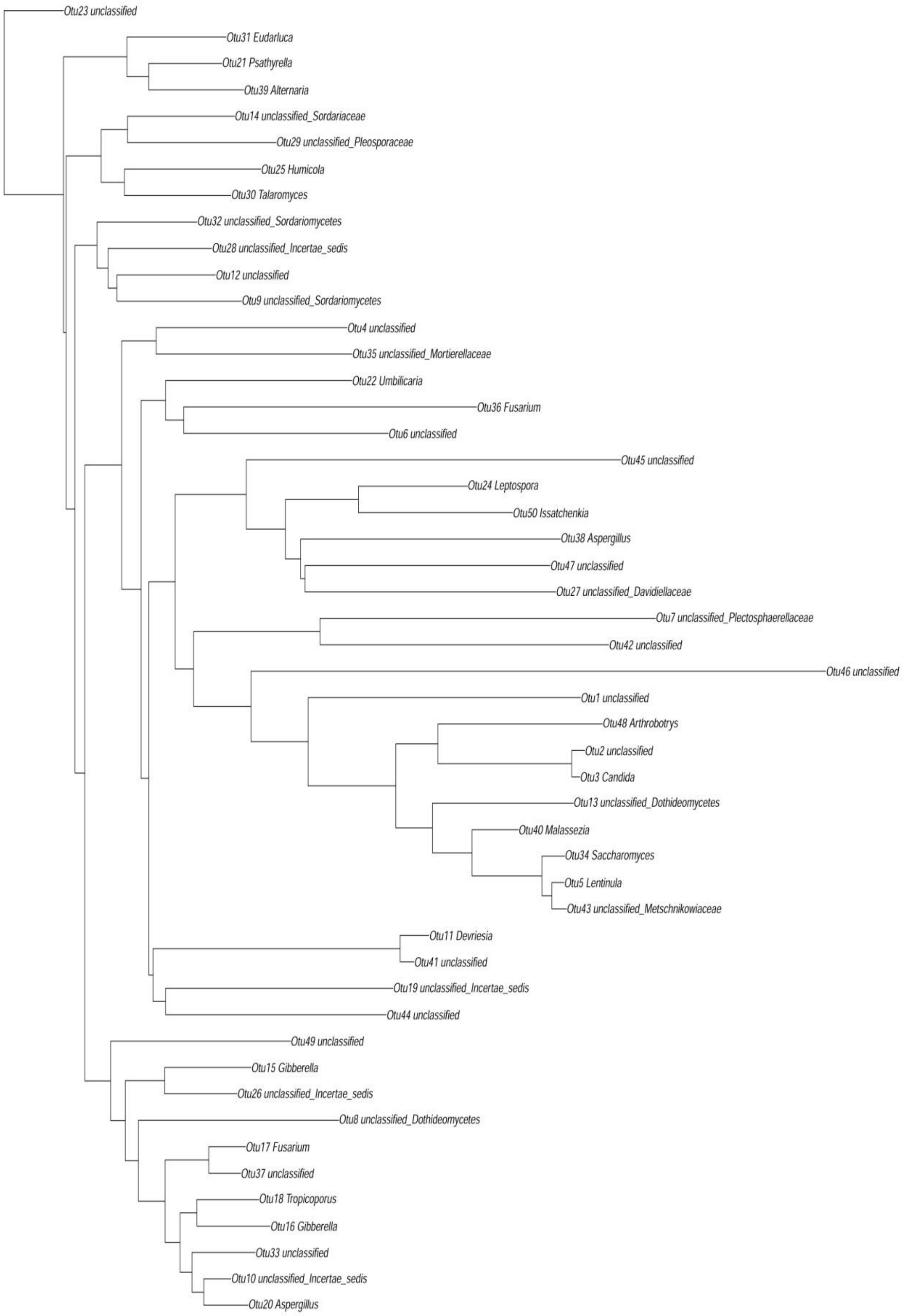

SUPPLEMENTARY FIGURE S2 Phylogenetic tree of representive of the first 50 OTUs based on ITS2 rRNA genes. This tree indicated the genetic relationship and difference of OTUs, and created by approximately-maximum-likelihood method phylogenetic trees through the FastTree software.

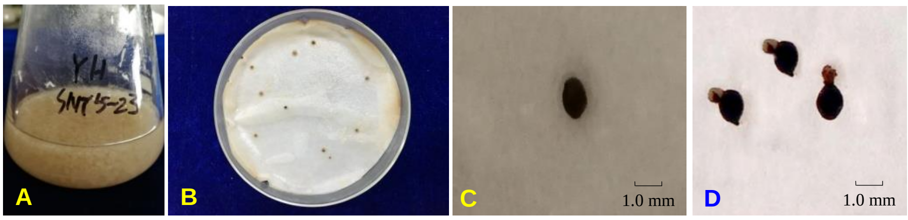

SUPPLEMENTARY FIGURE S3 The schedule of the test procedure of bio-activity test of endophytic fungi promoting *C. songaricum* seed germination *in vitro*. (A) *Fusarium redolen* (KY379544) incubated at 220 rpm on rotary shaker at 25 ℃ for 7 d; **(B)** The test of broth extract from *F. redolen* fermentation promoting seed germination; **(C)** The *C. songaricum* seed before germination; **(D)** The germination seeds promoted by *F. redolen* fermentation broth extract.
